# Home-based monitoring of cerebral oxygenation in response to postural changes using near-infrared spectroscopy

**DOI:** 10.1007/s11357-024-01241-w

**Published:** 2024-06-18

**Authors:** Marjolein Klop, Jurgen A. H. R. Claassen, Marianne J. Floor-Westerdijk, Richard J. A. van Wezel, Andrea B. Maier, Carel G. M. Meskers

**Affiliations:** 1 Department of Neurobiology, Donders Institute for Brain, Cognition and Behaviour, Radboud University, Nijmegen, The Netherlands; 2 Department of Geriatric Medicine, Radboud University Medical Center, Nijmegen, The Netherlands; 3 Department of Cardiovascular Sciences, University of Leicester, Leicester, UK; 4 Artinis Medical Systems, Elst, The Netherlands; 5 OnePlanet Research Center, Radboud University, Nijmegen, The Netherlands; 6 Department of Biomedical Signals and Systems, Technical Medical Centre, University of Twente, Enschede, The Netherlands; 7 Healthy Longevity Translational Research Program, Yong Loo Lin School of Medicine, National University of Singapore, Singapore, Singapore; 8 Centre for Healthy Longevity, @AgeSingapore, National University Health System, Singapore, Singapore; 9 Department of Human Movement Sciences, @AgeAmsterdam, Faculty of Behavioural and Movement Sciences, Amsterdam Movement Sciences, Vrije Universiteit Amsterdam, Amsterdam, The Netherlands; 10 Department of Rehabilitation Medicine, Amsterdam UMC Location Vrije Universiteit Amsterdam, Amsterdam Movement Sciences, Amsterdam, The Netherlands

**Keywords:** Ambulant monitoring, Near-infrared spectroscopy, Oxygenation, Orthostatic hypotension

## Abstract

Orthostatic hypotension (OH) is prevalent in older adults and can cause falls and hospitalization. Diagnostic intermittent blood pressure (BP) measurements are only a proxy for cerebral perfusion and do not reflect daily-life BP fluctuations. Near-infrared spectroscopy (NIRS)-measured cerebral oxygenation potentially overcomes these drawbacks. This study aimed to determine feasibility, face validity, and reliability of NIRS in the home environment. Ten participants with OH (2 female, mean age 77, *SD* 3.7) and 11 without OH (5 female, mean age 78, *SD* 6.7) wore a NIRS sensor at home on two different days for 10–11 h per day. Preceded by a laboratory-situated test, cerebral oxygenation was measured during three standardized supine-stand tests per day and during unsupervised daily life activities. Data availability, quality, and user experience were assessed (feasibility), as well as differences in posture-related oxygenation responses between participants with and without OH and between symptomatic (dizziness, light-headedness, blurred vision) and asymptomatic postural changes (face validity). Reliability was assessed through repetitive supine-stand tests. Up to 80% of the standardized home-based supine-stand tests could be analyzed. Oxygenation recovery values were lower for participants with OH (*p* = 0 .03–0.15); in those with OH, oxygenation showed a deeper maximum drop for symptomatic than asymptomatic postural changes (*p* = 0.04). Intra-class correlation coefficients varied from 0.07 to 0.40, with no consistent differences over measurements. This proof-of-concept study shows feasibility and face validity of at-home oxygenation monitoring using NIRS, confirming its potential value for diagnosis and monitoring in OH and OH-related symptoms. Further data are needed for conclusions about reliability.

**Supplementary Information:**

The online version contains supplementary material available at 10.1007/s11357-024-01241-w.

## Introduction

Orthostatic hypotension (OH) is prevalent among older adults, ranging from 6% in the general population [1] to 26% in adults aged 85 years or older [2]. The classic definition of OH is a prolonged blood pressure (BP) drop upon standing of at least 20 mmHg systolic and/or 10 mmHg diastolic, occurring within the first 3 min after assuming an upright posture [3]. OH can be accompanied by a decreased cerebral perfusion, causing symptoms like dizziness, light-headedness, diminished responsiveness, or tiredness when standing up [4, 5]. The association, either causal or as a marker of disease, of OH with falls [6], functional decline [7], dementia progression [8], cardiovascular disease [9], and mortality risk [10] underscores its clinical significance. Variability of the orthostatic response reduces the validity of a single measurement using a sphygmomanometer or oscillometric device to diagnose OH [11], and intermittent BP measurements do not capture fast changes in BP, hindering objectification of initial OH (OH occurring in the first 15 s of standing) [4]. Furthermore, BP drops after postural change often occur without clinical symptoms of OH, and vice versa [12], limiting the accuracy of clinical history to diagnose OH. Therefore, there is a need for assessment methods to be applied for longer durations, preferably at home.

Continuous BP monitoring using volume-clamp photoplethysmography, although non-invasive, is limited in measurement duration and mobility, for example, during movement or activity. This hampers reliable measurements at home [13–15]. Cuffless BP measurements with smartwatches lack accuracy in hypertensive and older participants [16], and performance to measure dynamical BP changes, e.g., during postural changes, is unknown [17]. Near-infrared spectroscopy (NIRS), measuring cerebral oxygenation through changes in oxygenated (O_2_Hb) and deoxygenated hemoglobin (HHb) in cerebral frontal lobe tissue, offers a potential alternative [18]. NIRS may better reflect orthostatic symptoms than BP due to its direct measurement of cerebral oxygenation [19]. Previous studies showed associations between BP and O_2_Hb dynamics, especially using long-channel NIRS, which seemed to capture part of the cerebral response to postural change [20, 21]. Yet, these studies were performed in a laboratory setting and did not include many participants with OH. Ambulatory (functional) NIRS was found to be feasible in healthy subjects during daily life, including postural changes and micturition [22], during flight in pilots [23], and during navigation in healthy young volunteers [24]. However, no prior research has explored at-home NIRS measurements for OH monitoring.

This study aimed to assess the feasibility (data availability, data quality, and user-friendliness), face validity (comparison OH/no OH and symptoms/no symptoms), and reliability (repeated measurements in the laboratory and over consecutive measurement days) of cerebral oxygenation monitoring during postural changes using NIRS at home.

## Methods

### Study design and participants

This prospective study was conducted at the Geriatric Outpatient Clinic of the Radboudumc in Nijmegen, the Netherlands, between February 2023 and January 2024. We recruited older (≥ 70 years) participants through flyers, advertisements, and registries of previous studies at the geriatric department with consent to be contacted for future research. Based on a screening laboratory visit, participants were enrolled in two groups: participants with OH and OH-related symptoms (“OH group”) and participants without OH and without severe OH-related symptoms in daily life or symptoms (“no OH group”). OH was defined as a drop in systolic BP of ≥ 20 mmHg and/or diastolic BP of ≥ 10 mmHg between 1 and 3 min after standing up using a 5-s moving average window [25], compared to the supine BP (1 min to 30 s before standing), measured with a continuous BP device (Finapres NOVA, Finapres Medical Systems, Enschede, the Netherlands), during at least one supine-stand transition. The presence of daily life symptoms was defined as reporting one or more OH-related symptoms, according to the OH symptom assessment (OHSA), and any impairment in daily life, according to the OH daily activity scale (OHDAS), defined severe symptoms [26]. Other inclusion criteria were the ability to provide informed consent and understand oral instructions and a functional ambulation category (FAC) score of at least 4 [27]. Participants were excluded when physically unable to perform supine-stand transitions, having moderate-to-severe dementia (clinical dementia rating ≥ 2 or Montreal Cognitive Assessment (MoCA) < 12), severely frail (clinical frailty scale ≥ 7) [28], or participating in an intervention study. The local ethics committee (CMO Radboudumc) concluded the study did not fall within the scope of the Medical Research Involving Human Subjects Act (WMO), thereby exempting the need for review by a central ethical committee. All participants signed written informed consent. The study was performed in accordance with the Declaration of Helsinki.

### Data collection

#### Screening laboratory visit

Participants were asked to complete The Older Persons and Informal Caregiver Survey-Short Form (TOPICS-SF) questionnaire about activities of daily living (ADL) and comorbidities [29] and the Technology Experience Profile (TEP) about technology use [30]. The MoCA was completed as a cognitive screening tool [31]. Moreover, information about age, height, weight, medication use (type and number of medications), alcohol use (units per week), smoking habits (yes/no), history of falls in the last year, and OH symptoms (OHSA and OHDAS) was obtained [26]. All participants performed a maximum grip strength, grip work (sustained grip strength), and five-times chair-stand test to indicate physical fitness. Participants performed one supine-stand transition (5 min supine; 3 min standing) while instructed to lie and stand still and perform the transition as fast as possible. Following the enrolment of 17 participants, this protocol was modified by involving 3 supine-stand transitions, to better account for the variability in orthostatic BP responses. BP was measured continuously using volume-clamp photoplethysmography on the digital artery of the left middle finger and intermittently (1 min before and 1 min and 3 min after standing up) using oscillometry on the contralateral brachial artery (Omron M4 Intelli IT, OMRON Healthcare, Kyoto, Japan). The hand wearing the Finapres device was placed in a sling to prevent hydrostatic pressure artifacts. Cerebral oxygenation was measured simultaneously by two NIRS sensors (PortaLite MkII, Artinis Medical Systems, Elst, The Netherlands) attached to the forehead bilaterally, approximately 2 cm above the eyebrows. The sensors consisted of three light-emitting diodes (LEDs) and two detectors, placed at inter-optode distances of 2.9, 3.5, and 4.1 cm (long channels) and 0.70, 0.80, and 0.74 cm (short channels). The NIRS sensor had an embedded inertial measurement unit (IMU) with triaxial accelerometry and gyroscope. Sensors were kept in place and covered by a black bandana to prevent ambient light interference. The control unit was placed in a belt around the waist.

#### At-home measurements

Measurements were performed at the participants’ homes on two different days, maximally 5 weeks apart. A measurement day started at 9:00 when the researcher visited the participant, and information was obtained about medication changes and falls since the previous visit. The researcher equipped the participant with the NIRS sensor, in the same way as during the screening visit, and a smartphone worn in a waist belt or pocket if possible. The smartphone contained an application (Krane™: the Orikami digital biomarker platform, Orikami, Nijmegen, the Netherlands) that guided participants in performing the supine-stand tests, registered the time stamps, and asked for OH-related symptoms. The NIRS sensors and phone application are depicted in Supplementary Material Fig. S1. Two ActivPALs (PAL technologies, Glasgow, Scotland) were placed on the thigh and rib to measure the participant’s posture (supine, sitting, standing, or stepping). The researcher explained the use of the NIRS sensor and phone application and turned all devices on. During a measurement day, participants performed three supine-stand tests (5 min supine; 3 min standing) spread over the day. The first of these was supervised by the researcher, who left the home afterwards; the remainder of the day was unsupervised, including the second and third repetition of the supine-stand tests. Occurrence of OH-related symptoms throughout the day was recorded in a diary and by pressing the event button on the NIRS device (see Fig. S1), after which the smartphone application asked for the type and severity of the symptoms and during what activity they occurred. At 20:00, participants could remove and turn off the NIRS sensors, ActivPALs, and smartphone. No other interference with the equipment, including charging of the batteries, was required during the day. The next day, the researcher picked up the equipment, and the system usability scale (SUS) was asked to be completed, together with questions for feedback, user comfort, and user-friendliness of the NIRS device and application [32].

### Data acquisition and processing

#### Screening laboratory visit

Cerebral oxygenation signals were acquired in OxySoft (version 3.4.12) at a sampling frequency of 100 Hz. BP signals were acquired in Acqknowledge at 200 Hz (version 5.0, BioPac Systems Inc., Goleta, USA). Both acquisition programs were synchronized using analog pulses (PortaSync MkII, Artinis Medical Systems, Elst, the Netherlands). BP and NIRS data were processed in MATLAB (2022a, MathWorks Inc., Natick, USA) using custom-written semiautomatic scripts [33]. Signal quality was assessed visually, and signals of insufficient quality were discarded. For BP, insufficient quality was defined as inability to visually distinguish any peaks and troughs. For cerebral oxygenation, the signal quality index (SQI) was used, rating signal quality between very bad (1) and very good (5) based on the presence of a heartbeat wave [34]. Channels were excluded when the average SQI was below 3. This threshold was set empirically. Sensitivity analyses to evaluate the effect of SQI threshold on the obtained results were performed using thresholds of respectively 3.5, considered good in resting situations [34], and 2, previously used at the neonatal ICU, a setting even more prone to artifacts [35]. In addition, signals were discarded when there was flatlining, a baseline shift larger than 10 µM, or an irregular amplitude. Heart rate, systolic BP (SBP), and diastolic BP (DBP) were obtained over time by peak and trough detection. BP, heart rate, and cerebral oxygenation signals were resampled at 10 Hz and filtered using a 5-s moving average filter [25]. All available long NIRS channels measuring cerebral oxygenation were averaged, while the short channels with an inter-optode distance of 0.80 cm of both sides were averaged when available.

#### At-home measurements

Cerebral oxygenation signals were acquired offline at a sampling frequency of 100 Hz, stored locally on the NIRS device, and transferred to MATLAB. Cerebral oxygenation signals were resampled at 10 Hz, filtered using a 5-s moving average filter, and linearly detrended to remove slow drift. Measurement starting time was retrieved retrospectively from the offline data. Oxygenation data were used in the analyses when they fulfilled the same criteria as for the screening measurement.

ActivPAL data were stored on the device and offline transferred to Microsoft Excel (Office 16) using PALconnect before processing in MATLAB. ActivPAL and NIRS/IMU data were synchronized by clock times at the start of the measurement. The signal vector magnitude (from now on called “total acceleration”) of the triaxial accelerometer signal was calculated by $\sqrt{{x}^{2}+{y}^{2}+{z}^{2}}$, with acceleration *x* in the vertical (superior-inferior) direction, acceleration *y* in the horizontal (left–right) direction, and acceleration *z* in the horizontal (anterior–posterior) direction [36]. Total acceleration was band-pass filtered (0.1–1.3 Hz cutoff, zero-phase second-order Butterworth filter) to remove high-frequency noise and slow baseline drifts.

### Data analysis

#### Feasibility

Average user comfort and SUS scores were calculated for both measurement days separately. The SQI was determined over each supine-stand repetition and each of the entire measurement days.

#### Supine-stand tests in the laboratory and at home

Exact times of the standardized tests at home were retrieved from the data platform linked to the smartphone application and, if necessary, corrected using NIRS-device accelerometer data. Within these time frames, accelerometer (obtained by NIRS sensor) and cerebral oxygenation data were reviewed, likewise to the accelerometer, cerebral oxygenation, and BP data as measured during the test at the laboratory. Cerebral oxygenation outcome parameters (O_2_Hb) were determined relative to baseline values defined as the average at 60 to 30 s before standing up: (1) maximum drop amplitude and recovery respectively at (2) 30–40 s (early), (3) 50–60 s (1 min), and (4) 60–170 s (late) after standing up.

#### Daily activities and daily postural changes

The unsupervised and uncontrolled postural changes were identified via the ActivPAL readouts. The transition was classified as a sit-stand when the leg sensor code changed from sitting to standing or sitting to stepping. In a window of 40 s around the ActivPAL-detected sit-stand transition, the highest peak of the filtered total acceleration was identified and assumed to be the exact sit-stand transition. The cerebral oxygenation and total accelerometer curves were extracted 30 s before the detected postural change and 1 min after the detected postural change and determined relative to the sitting baseline (45 to 15 s before standing). Signals with a minimum–maximum long-channel oxygenation difference larger than 20 µmol/L were discarded as being physiologically improbable.

#### OH-related symptoms

For each reported event, possible cerebral oxygenation drops and postural changes (IMU data) were identified from the prior 5 to the succeeding 5 min.

### Statistical analysis

Statistical analyses were performed in MATLAB (R2022a), RStudio (2022.02.1, R version 4.1.3), and IBM SPSS Statistics 29. All continuous variables are presented as mean (standard deviation) when normally distributed or as median (interquartile range) when distributed otherwise. Categorical variables are presented as number (percentage). We used two-sided testing with a significance level of 0.05 for all analyses.

Three linear mixed models were created for each outcome parameter (maximum drop amplitude, early recovery, 1-min recovery, and late recovery) of the long- and short-channel oxygenation during a supine-stand transition. Model 1 used fixed effects for group (“OH” or “no OH”) and condition (“laboratory” or “at home”) and random effects for participants. Model 2 included only OH participants (random effects) and used fixed effects for symptoms (“yes” or “no”) and condition (“laboratory” or “at home”). Model 3 included only the at-home condition, separated into “day 1” and “day 2,” and the repetitions within a measurement day (“repetition 1,” “repetition 2,” or “repetition 3”), with random effects for participants. In all models, when interaction effects were nonsignificant, the simplest model without interaction effects was used. Reliability was represented by the two-way mixed-effect intraclass correlation coefficient (ICC) determined from all models, expressing the proportion of total variance attributed to the between-subject variability.

## Results

### Baseline characteristics

**Fig. 1:**
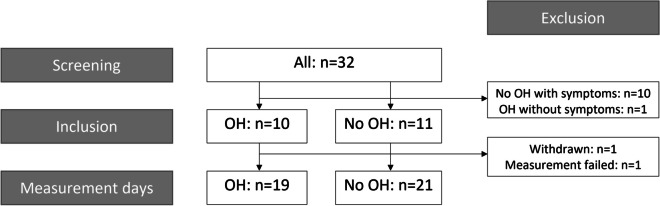
Participant flow. OH, orthostatic hypotension

Thirty-two older adults were screened for eligibility. Ten were classified in the “OH group” and 11 in the “no OH” group, of whom one was withdrawn due to personal circumstances after one measurement day and was therefore replaced. One potential participant was excluded due to OH without symptoms and 10 due to severe OH-related symptoms, such as dizziness, in the absence of OH during the screening laboratory visit (see Fig. 1). Participants’ baseline characteristics are shown in Table 1. Daily life OH symptoms reported most frequently by the participants with OH were dizziness/light-headedness, reported by all, and blurred vision, reported in half.

**Table 1:** Baseline characteristics for participants with and without OH, presented as mean (standard deviation (SD)) or median (interquartile range (IQR)) for continuous variables and number (percentage) for categorical variables

Characteristic	With OH (*n* = 10)	Without OH (*n* = 11)
Age (years), mean (SD)	77 (3.9)	78 (6.7)
Sex, female	2 (20)	5 (45)
BMI (kg/m^2^), mean (SD)	24 (3.1)	25 (3.0)
Smoking	0 (0)	0 (0)
Excessive alcohol use^a^	0 (0)	0 (0)
History of CVD^b^	4 (40)	4 (36)
Diabetes mellitus	1 (10)	2 (18)
OH symptoms during measurements	5 (50)	0 (0)
Any OH symptoms in daily life^c^	10 (100)	3 (27)
Dizziness, light-headedness	10 (100); 4.7 (1.6)	3 (27); 2.7 (1.2)
Blurred vision	5 (50); 6.2 (3.1)	0 (0)
Muscle weakness	3 (33); 4.3 (1.5)	0 (0)
Fatigue	2 (20); 7 (0)	0 (0)
Concentration problems	1 (10); 5 (0)	1 (9); 2 (0)
Head/neck ache	2 (20); 3.5 (2.1)	0 (0)
Severe OH symptoms in daily life^d^	7 (70)	0 (0)
Falls in last year	7 (70)	6 (55)
Number of falls, median (IQR)	1 (7)	1 (1)
MoCA, mean (SD)	25 (3.5)	27 (1.4)
Medication use	9 (90)	10 (91)
Antihypertensive drug use	6 (60)	8 (73)
Statin use	5 (50)	8 (73)
Antidepressant use	2 (20)	1 (9)
Systolic BP (mmHg), mean (SD)	145 (20)	151 (11)
Diastolic BP (mmHg), mean (SD)	81 (7.5)	82 (8)
Maximum grip strength (kPa), mean (SD)	67 (20)	55 (22)
Grip work (kPa s), mean (SD)	2124 (1740)	2612 (2754)
Chair-stand test (s), mean (SD)	11.2 (3.8)	10.5 (2.6)
Technology use score, mean (SD)^e,f^	119 (15)	143 (8.8)

^a^Excessive alcohol use: more than 14 units per week for females and more than 21 units per week for males. ^b^In the last 12 months as reported by the TOPICS-SF questionnaire. ^c^Reported as number (percentage) and mean (SD) where mean (SD) represents the mean score on a severity scale from 1 (very mild symptoms) to 10 (very severe symptoms). ^d^Defined as orthostatic hypotension (OH) symptoms limiting standing or walking for a short (< 1 min) or longer (> 1 min) period of time. ^e^Score based on technology experience profile (TEP) [30]. Older adults have been divided into groups based on their TEP score before as novice (scoring 36–54), beginner (72–90), competent (108–126), and proficient (144–162) [37]. The lowest TEP in our sample was 102, scoring between beginner and competent. ^f^Missing values (4 items from 3 participants) were imputed based on answers to other questions (e.g. when someone reported to use productivity software, computer use was also assumed). *MoCA*, Montreal Cognitive Assessment; *CVD*, cardiovascular disease; *BMI*, body mass index

### Feasibility

#### Data availability

Data from one measurement day of one participant were lost due to technical issues (Fig. 1). SQI was, on average, 3.6 (*SD* 0.9) for the long NIRS channels and 3.7 (1.0) for the short channels. Fig. S2 shows the SQI during the different repetitions, showing both a decline and improvement over time. Movement artifacts, low light intensity (e.g., caused by a hair in front of an LED), or ambient light artifacts caused low SQI scores. For example, being outside on a sunny day appeared to cause flatlining in the long NIRS channels due to too much ambient light, despite the use of the black bandana covering the unit. Every participant was able to perform supine-stand tests in the laboratory and at home; eight supine-stand tests were missing for various reasons (Table 2). During the standardized tests at home (from 1 min before to 3 min after standing up), the SQI was on average 4.0 (1.1) for long and 3.9 (1.1) for short channels. Data from 32 (13%) long channels and 65 (27%) short channels had to be excluded due to insufficient data quality (see Table 2). Two participants were unable to use the mobile phone application due to technical issues; the paper diary was then used as backup.

**Table 2:** NIRS data availability and quality during standardized supine-stand tests. In total, there were 58 laboratory recordings (2 sensors measuring 17 participants once and 4 participants three times (after amendment)) and 240 at-home recordings (2 sensors measuring three times during 40 measurement days of 21 participants). Data are presented as number of recordings (percentage) when not indicated otherwise

Condition	Laboratory (*n* = 58)		At home (*n* = 240)	
Channel	Long	Short	Long	Short
Absent	0 (0)	0 (0)	16 (7)	16 (7)
Accidental discontinuation	-	-	8 (3)	8 (3)
Performed after removing sensor	-	-	2 (1)	2 (1)
Lost connection between sensor and application	-	-	2 (1)	2 (1)
Unknown (not performed)	-	-	4 (2)	4 (2)
Recordings discarded	0 (0)	6 (10)	32 (13)	65 (27)
SQI (mean (SD))	4.6 (0.4)	4.6 (0.7)	4.0 (1.0)	3.9 (1.1)
SQI < 3	0 (0)	4 (7)	25 (10)	50 (21)
Large baseline shift (> 10 µM)	0 (0)	2 (3)	6 (3)	14 (6)
Irregular amplitude	0 (0)	0 (0)	0 (0)	1 (0)
Sensitivity analyses (supplementary material)				
SQI < 2	0 (0)	1 (2)	14 (6)	22 (9)
Large baseline shift (> 10 µM)	0 (0)	2 (3)	8 (3)	18 (8)
Irregular amplitude	0 (0)	0 (0)	0 (0)	1 (0)
SQI < 3.5	3 (5)	5 (9)	48 (20)	64 (27)
Large baseline shift (> 10 µM)	0 (0)	2 (3)	3 (1)	10 (4)
Irregular amplitude	0 (0)	0 (0)	0 (0)	1 (0)

*SQI* signal quality index. Signal exclusion was only identified as due to irregular amplitude or large baseline shift when signal was not already excluded because of low SQI

#### User experience and comfort

All participants were able to wear the NIRS sensors for the entire day. Some participants experienced a headache after wearing the sensor for several hours with relief after moving the sensor under the bandana. On average, participants rated sensor comfort with 7.6 (*SD* 1.2) out of 10 during the first measurement day and 6.6 (1.9) during the second. For 6 (out of 21; first measurement day) to 12 (out of 20; second measurement day) participants, wearing comfort changed over time, mainly worsening. Four (out of 21; first day) and 5 (out of 20; second day) participants felt hindered by the sensor, mainly due to visibility of the black bandana over the forehead, irritating cables, or discomfort. The average SUS score for the combination of sensor and mobile phone application was 72 (out of maximally 100; *SD* 15; *n* = 21, first day) and 69 (16; *n* = 20, second day). The statement with the lowest score was “I think that I would like to use this system frequently,” and the statement scoring highest was ‘I found the system unnecessarily complex.” A specific issue encountered in using the application was a lost Bluetooth connection when phone and sensor were too far apart. The result was that the participant could no longer answer questionnaires on the phone, which was solved by writing down the answers on paper.

### Face validity

#### Supine-stand tests in the laboratory and at home

**Fig. 2:**
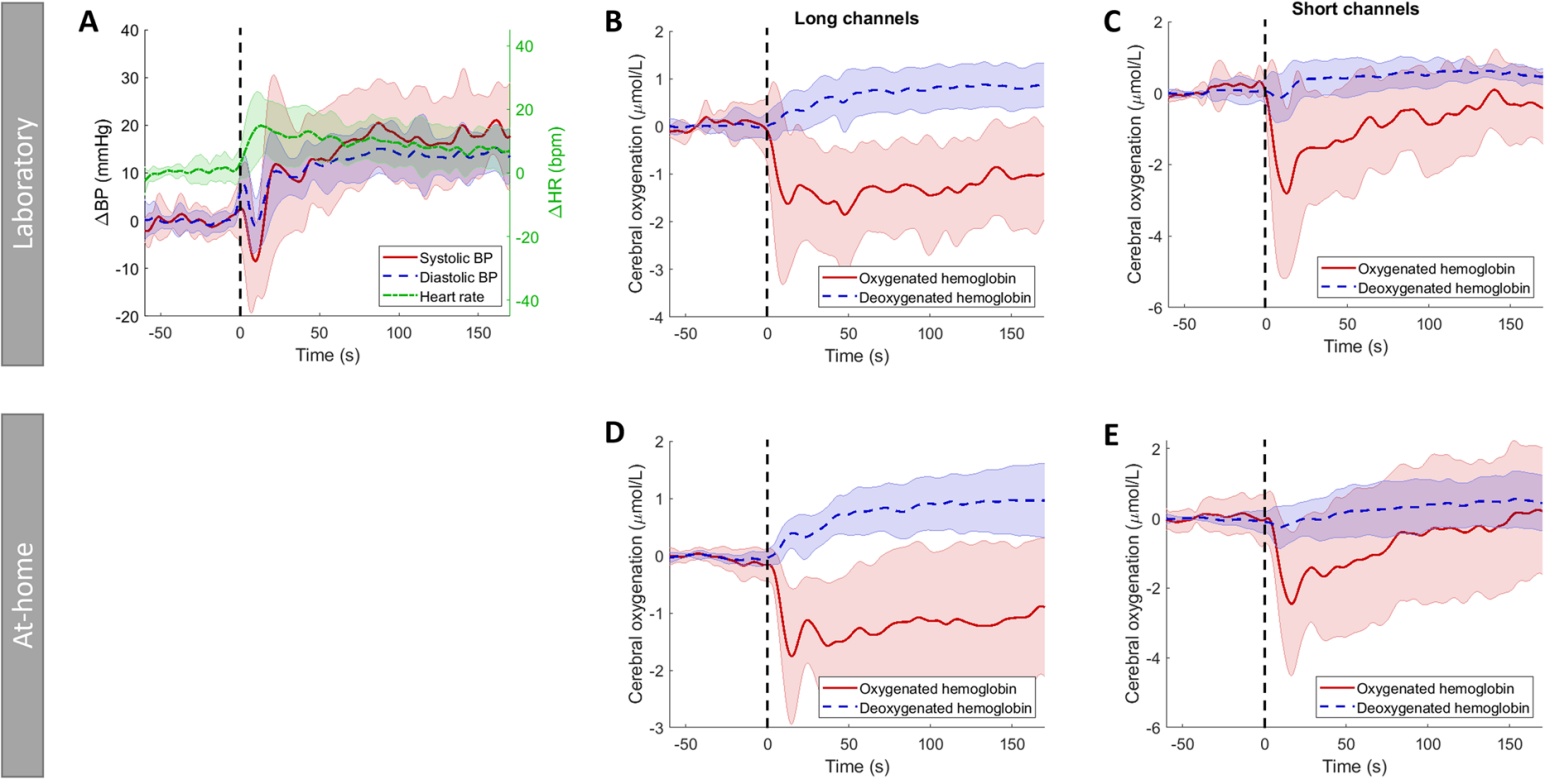
Standardized supine-stand test for the participants without orthostatic hypotension (OH) in the laboratory (**A**, **B**, **C**) and at home (**D**, **E**), showing responses of **A** blood pressure (BP), systolic in red (solid line), diastolic in blue (dashed line), and heart rate (HR) in green (dashed-dotted line). **B** + **D** Cerebral oxygenation measured with long channels, oxygenated hemoglobin in red, and deoxygenated hemoglobin in blue (dashed line) and **C** + **E** cerebral oxygenation measured with short channels, oxygenated hemoglobin in red, and deoxygenated hemoglobin in blue (dashed line). All signals are shown from 1 min before standing up to 170 s after standing up. These are averaged over all repetitions and subjects without OH (laboratory measurements) or all repetitions, measurement days, and subjects without OH. Standing up is indicated by a vertical black dashed line. Shaded areas show standard deviations for all signals

**Fig. 3:**
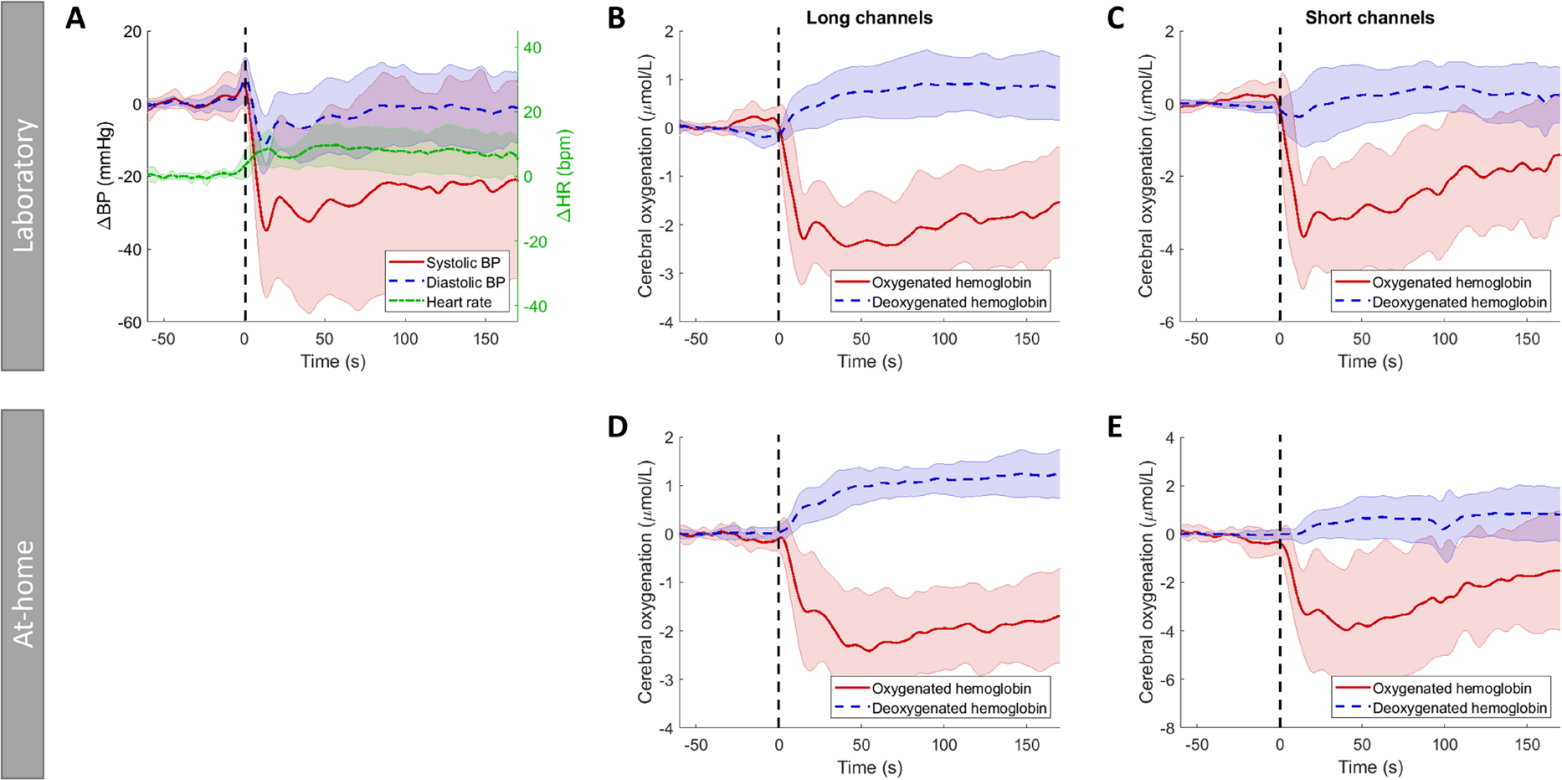
Standardized supine-stand test for the participants with orthostatic hypotension (OH) in the laboratory (**A**, **B**, **C**) and at home (**D**, **E**), showing responses of **A** blood pressure (BP), systolic in red (solid line), diastolic in blue (dashed line), and heart rate (HR) in green (dashed-dotted line). **B** + **D** Cerebral oxygenation measured with long channels, oxygenated hemoglobin in red, and deoxygenated hemoglobin in blue (dashed line) and **C** + **E** cerebral oxygenation measured with short channels, oxygenated hemoglobin in red, and deoxygenated hemoglobin in blue (dashed line). All signals are shown from 1 min before standing up to 170 s after standing up. These are averaged over all repetitions and subjects with OH (laboratory measurements) or all repetitions, measurement days, and subjects with OH. Standing up is indicated by a vertical black dashed line. Shaded areas show standard deviations for all signals

Figure 2 shows the average responses of the laboratory supine-stand tests (A, B, C), the averaged at-home supine-stand tests (D, E), and averaged overall participants without OH and Fig. 3 overall participants with OH. Laboratory and at-home supine-stand tests resulted in a comparable oxygenation curve. Standing up caused a drop in O_2_Hb, followed by a gradual recovery, whereas long-channel HHb slightly increased after standing up. Having OH gave lower O_2_Hb (30–170 s) recovery values than not having OH, especially for the short channels (*p* = 0.026–0.032; long channel: *p* = 0.063–0.145; Table 3).

**Table 3:** Results of mixed model 1 during standardized supine-stand tests with participants as random effects and group (“OH” (modeled as 1) or “no OH” (modeled as 0)) and condition (“laboratory” (modeled as 0) or “at home” (modeled as 1)) as fixed effects on long- or short-channel oxygenation (O_2_Hb) parameters (maximum drop amplitude, early recovery (30–40 s after postural change), 1-min recovery (50–60 s after postural change), and late recovery (60–170 s after postural change)). *n* is the total number of supine-stand transitions (number of subjects of whom these supine-stand transitions are included)

O_2_Hb long	Fixed effects group		Fixed effects condition		Random effects	
*n* = 132 (21)	Estimate (95% *CI*)	*p*-value	Estimate (95% *CI*)	*p*-value	Variance	ICC
Maximum drop	− 0.15 (− 1.03–0.73)	0.728	0.30 (− 0.20–0.80)	0.231	0.69	0.33
Early recovery	− 0.54 (− 1.27–0.20)	0.145	0.34 (− 0.15–0.82)	0.172	0.43	0.25
1-min recovery	− 0.84 (− 1.73–0.05)	0.063	0.25 (− 0.321–0.81)	0.389	0.65	0.27
Late recovery	− 0.80 (− 1.72–0.12)	0.085	0.17 (− 0.37–0.71)	0.542	0.74	0.32
O_2_Hb short *n* = 119 (21)						
Maximum drop	− 1.21 (− 3.03–0.61)	0.179	− 0.32 (− 1.40–0.76)	0.563	2.85	0.33
Early recovery	− 1.82 (− 3.41 to − 0.24)	0.026*	− 0.21 (− 1.25–0.82)	0.686	2.00	0.27
1-min recovery	− 2.18 (− 4.14 to − 0.21)	0.032*	− 0.54 (− 1.80–0.71)	0.389	3.14	0.29
Late recovery	− 1.92 (− 3.59 to − 0.25)	0.026*	0.03 (− 1.33–1.39)	0.964	1.58	0.15

#### Daily activities and daily postural changes

**Fig. 4:**
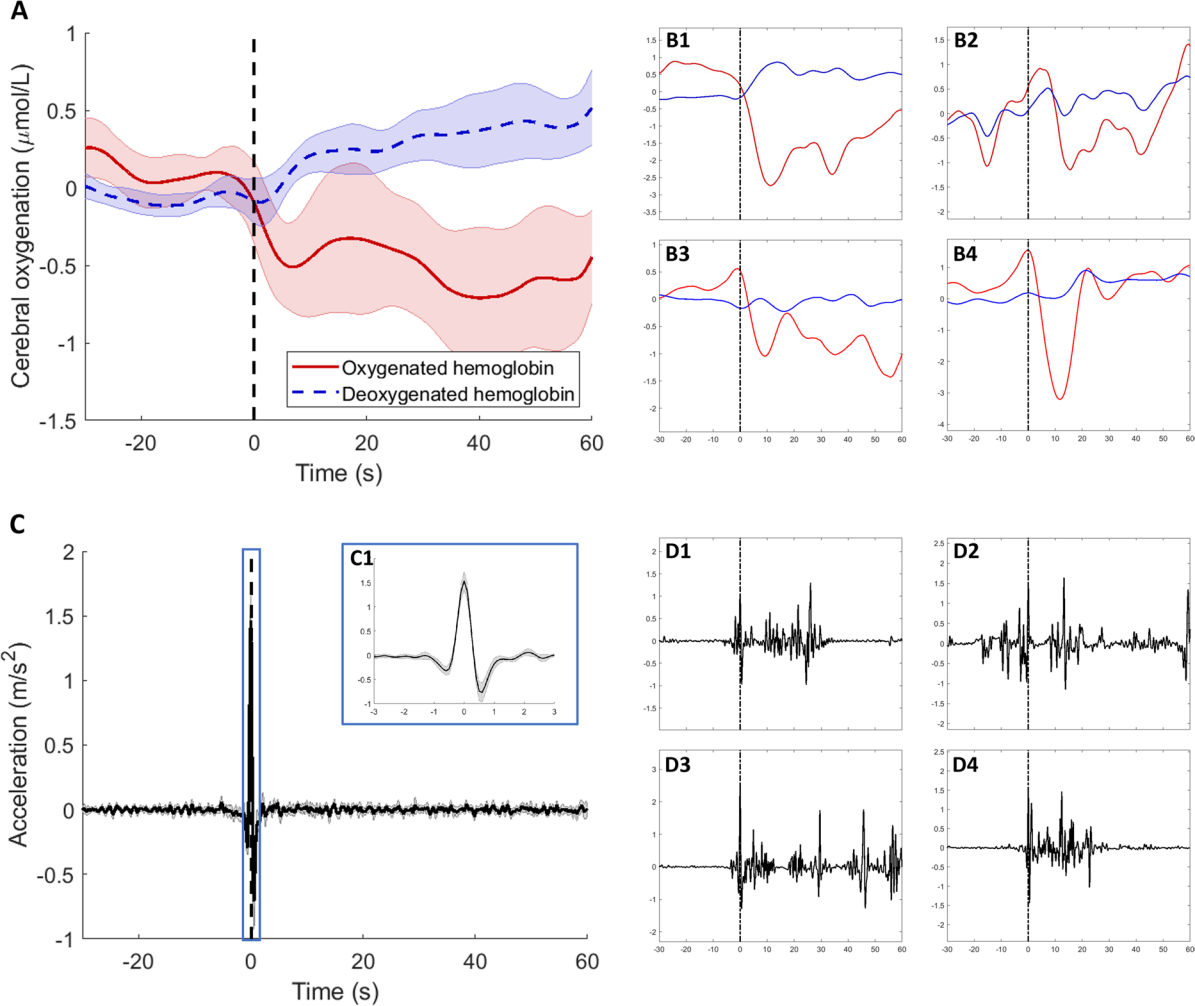
Spontaneous daily sit-stand transitions averaged over all participants with and without orthostatic hypotension (OH), with long-channel oxygenation (oxygenated hemoglobin in red, deoxygenated hemoglobin in blue) in **A** + **B** and total acceleration in **C** + **D**. All data are shown from 30 s before postural change (as detected by ActivPALs and corrected by accelerometer signals) to 1 min after postural change. This sit-stand transition is indicated by the black dashed line. C1 is a zoomed-in version of the acceleration signal showing 3 s before to 3 s after postural change. B1 and D1 show a sit-stand transition of a participant with severe OH, B2 and D2 show a noisy sit-stand transition, B3 and D3 show multiple sit-stand transitions within a short period of time, and B4 and D4 show a clear sit-stand transition

During an average measurement day, participants had a metabolic equivalent of task (MET) score of 1.5 (*SD* 0.1), with 38 (9) postural changes. Sit-stand transitions could be identified using the combination of ActivPALs and accelerometers from the NIRS device. On average, a daily life sit-stand transition resulted in a maximum drop of − 0.8 µmol/L in long channels and − 1.1 µmol/L in short channels compared to the seated baseline value (Fig. 4A). The acceleration pattern showed an average initial rise to 1.5 m/s^2^ followed by a drop to − 0.8 m/s^2^, within 2 s (Fig. 4C and panel C1). Responses to a daily postural change were variable. Examples are shown in Fig. 4, e.g., a clear drop in someone with severe OH (panel B1 and panel D1), no clear change with a noisy accelerometer signal (panels B2 and D2), multiple sit-stand-like acceleration patterns (panels B3 and D3), and a clear sit-stand transition (panels B4 and D4).

#### OH-related symptoms

None of the participants without OH experienced symptoms during the measurement days. Five OH participants experienced symptoms during the first and three also during the second measurement day. Four participants with OH only experienced symptoms during the standardized supine-stand tests, while one experienced symptoms on other occasions, i.e., two episodes with symptoms during the first and one during the second measurement day. No clear oxygenation drop or postural change-related accelerometer pattern could be identified (Fig. S4 in the supplementary material). In total, 21 of 78 standardized supine-stand tests in the laboratory and at home were symptomatic. Within OH participants, the symptomatic supine-stand tests induced a larger O_2_Hb drop, measured with both long and short channels (*p* = 0.042 and *p* = 0.039, respectively, Table 4), whereas O_2_Hb recovery values did not differ significantly between symptomatic and asymptomatic standardized postural changes (*p* > 0.119). The oxygenation course during symptomatic versus asymptomatic postural changes is shown in Fig. S3 in the supplementary material.

**Table 4:** Results of mixed model 2 during standardized supine-stand tests in the subgroup of participants with OH with participants as random effects and group (“symptomatic” (modeled as 1) or “asymptomatic” (modeled as 0)) and condition (“laboratory” (modeled as 0) or “at home” (modeled as 1)) as fixed effects on long- or short-channel oxygenation (O_2_Hb) parameters (maximum drop amplitude, early recovery (30–40 s after postural change), 1-min recovery (50–60 s after postural change), and late recovery (60–170 s after postural change)). *n* is the total number of supine-stand transitions (number of subjects of whom these supine-stand transitions are included)

O_2_Hb long	Fixed-effects group		Fixed-effects condition		Random effects	
*n* = 69 (10)	Estimate (95% CI)	p-value	Estimate (95% CI)	p-value	Variance	ICC
Maximum drop	− 0.80 (− 1.57 to − 0.03)	0.041*	0.33 (− 0.35–1.01)	0.334	0.60	0.32
Early recovery	− 0.38 (− 1.17–0.41)	0.338	0.36 (− 0.36–1.08)	0.324	0.33	0.18
1-min recovery	− 0.19 (− 1.12–0.74)	0.683	0.16 (− 0.69–1.01)	0.708	0.39	0.16
Late recovery	− 0.66 (− 1.51–0.18)	0.121	0.07 (− 0.71–0.84)	0.861	0.36	0.17
O_2_Hb short *n* = 65 (10)						
Maximum drop	− 1.75 (− 3.40 to − 0.09)	0.039*	− 0.94 (− 2.40–0.51)	0.198	2.04	0.26
Early recovery	− 1.51 (− 3.22–0.20)	0.083	− 0.69 (− 2.20–0.82)	0.363	2.01	0.25
1-min recovery	− 1.19 (− 3.40–1.02)	0.287	− 1.06 (− 3.02–0.90)	0.282	3.03	0.23
Late recovery	− 1.45 (− 3.68–0.76)	0.193	− 0.41 (− 2.49–1.67)	0.691	0.90	0.07

### Reliability

Oxygenation parameters derived from standardized supine-stand tests at home did not differ significantly from those retrieved from the same tests in a laboratory setting (Table 3, *p* > 0.170). ICCs for repeated supine stands in the laboratory and at home ranged from 0.25 (early recovery) to 0.33 (maximum drop amplitude) for long-channel O_2_Hb and 0.15 (late recovery) to 0.33 (maximum drop amplitude) for short-channel O_2_Hb (Table 3). O_2_Hb outcome parameters did not differ significantly (*p* > 0.133) between the two at-home days and between the three repetitions during a measurement day, except for the short-channel O_2_Hb late recovery, having lower values for repetition 2 (*p* = 0.04, Table 5). All ICCs were low (*ICC* < 0.40).

**Table 5:** Results of mixed model 3 during standardized supine-stand tests in the subgroup of at-home measurements with participants as random effects and repetition (1 (modeled as 0), 2 (modeled as 1), or 3 (modeled as 2)) and condition (“at-home day 1” (in model as 0) or “at-home day 2” (in model as 1)) as fixed effects on long- or short-channel oxygenation (O_2_Hb) parameters (maximum drop amplitude, early recovery (30–40 s after postural change), 1-min recovery (50–60 s after postural change), and late recovery (60–170 s after postural change)). *n* is the total number of supine-stand transitions (number of subjects of whom these supine-stand transitions are included). *Indicates significant differences

O_2_Hb long	Fixed effects Repetition 2		Repetition 3		Condition		Random effects	
*n* = 103 (21)	Estimate (95% *CI*)	*p*-value	Estimate (95% *CI*)	*p*-value	Estimate (95% *CI*)	*p*-value	Variance	ICC
Maximum drop	− 0.16 (− 0.70–0.39)	0.571	0.09 (− 0.50–0.68)	0.767	− 0.05 (− 0.52–0.43)	0.844	0.84	0.38
Early recovery	− 0.17 (− 0.71–0.37)	0.532	− 0.03 (− 0.61–0.56)	0.928	0.12 (− 0.35–0.59)	0.625	0.64	0.32
1-min recovery	− 0.21 (− 0.85–0.43)	0.513	− 0.02 (− 0.72–0.67)	0.945	0.11 (− 0.45–0.67)	0.699	1.04	0.35
Late recovery	− 0.41 (− 1.04–0.22)	0.199	− 0.19 (− 0.87–0.49)	0.575	0.40 (− 0.15–0.95)	0.149	0.97	0.34
O_2_Hb short *n* = 92 (21)								
Maximum drop	− 1.18 (− 2.37–0.01)	0.052	− 0.63 (− 1.91–0.64)	0.325	0.34 (− 0.72–1.39)	0.524	3.99	0.41
Early recovery	− 0.78 (− 1.97–0.41)	0.195	− 0.73 (− 2.00–0.54)	0.258	0.12 (− 0.94–1.17)	0.826	3.57	0.38
1-min recovery	− 0.87 (− 2.33–0.58)	0.235	− 0.95 (− 2.51–0.61)	0.230	− 0.07 (− 1.36–1.22)	0.915	5.65	0.40
Late recovery	− 1.65 (− 3.27 to − 0.03)	0.047*	− 1.26 (− 3.00–0.47)	0.152	0.76 (− 0.66–2.19)	0.289	2.82	0.21

## Discussion

### Main findings

In this study, feasibility, validity, and reliability of NIRS for capturing oxygenation responses to standardized supine-stand and non-standardized daily postural changes in the home environment were established to support diagnosis and monitoring of OH. OH and non-OH participants were able to follow the assigned protocol; user experience and acceptability were sufficient, and measuring at home was feasible. Data quality strongly varied between individuals; 13% (long channels) to 27% (short channels) of the standardized supine-stand tests had to be discarded due to artifacts. During standardized supine-stand tests, participants with OH tended to have lower oxygenation recovery (30–180 s after postural change) values (*p* = 0.06–0.15, depending on the timepoint, in long channels, *p* = 0.03 in short channels) than participants without OH. Within subjects with OH, postural changes accompanied by symptoms like dizziness, light-headedness, or blurred vision showed a significantly larger oxygenation maximum drop than asymptomatic postural changes. Daily postural changes (sit-stand), identified by accelerometer signals, resulted, on average, in a drop in oxygenation but showed much variability within but also between subjects. Laboratory and at-home standardized supine-stand oxygenation measurements did not differ significantly, similar to measurements during two different days at home. Repeated supine-stand tests showed low intra-class correlations (0.17–0.40).

### Feasibility

Participants reported a sufficient user experience and overall acceptance. The SUS score of 69 to 72 is around the threshold of 70 for acceptable usability [38]. As some questions were not in line with the intended incident use of the NIRS sensor (e.g., “I think that I would like to use this system frequently,” which rated lowest), we consider this score acceptable. Rated user comfort differed per individual, like data quality, and these two feasibility aspects are strongly related. There is a positive relationship between pressure on the head and data quality and most often a negative between pressure on the head and wearing comfort [39]. However, some participants in our study reported discomfort due to a too-loose fit of the bandana and sensor movement (thus low pressure). Insufficient data quality in our study was caused by motion artifacts, too much ambient light and low light intensities, similar to previous studies in free-living conditions, as summarized in a review article [40]. We chose not to exclude channels with small motion artifacts and not to correct these as these were hard to distinguish from physiological oxygenation changes. At a group level, small motion artifacts proved not to be a problem (shown by sensitivity analyses using other SQI thresholds for channel exclusion). However, at individual level, a motion artifact appeared to alter the captured, but not the real, oxygenation response to a postural change. Motion artifacts should, therefore, be prevented as much as possible and could have been diminished by using skin-friendly stickers to attach the sensor. We chose not to, due to vulnerable older skin and to keep the possibility of changing sensor position. The latter was an advantage in participants with a headache after a few hours of sensor wear. When the bandana fitted well in this study, data quality could be excellent over almost the entire measurement day. A tailored solution for sensor attachment might help to improve data quality and wearing comfort.

### Face validity

Cerebral oxygenation responses to standardized supine-stand tests at home followed a similar course as tests performed in the laboratory in mostly healthy younger and older adults [21, 41]. This study revealed lower oxygenation recovery values for older adults with OH. These were more profound in short channels measuring superficial skin and scalp perfusion than in long channels also capturing cerebral perfusion. This corresponds to the expected effect of cerebral autoregulation attenuating the impact of a BP drop on CBF [42], which would result in larger drops in skin perfusion than in brain perfusion. Lower oxygenation recovery values are as expected because OH is defined as impaired BP recovery, and BP and oxygenation are correlated [21]. Within the OH group, we observed large differences in oxygenation response, but OH severity in terms of BP recovery also differed. OH was not consistently present during all postural changes within a participant, and often not symptomatic. When distinguishing symptomatic from asymptomatic supine-stand transitions within participants with OH, a deeper oxygenation maximum drop was found during symptomatic postural changes, indicating that the lowest oxygenation value might be more relevant for symptom experience than time to recovery. This was not found in a large cohort of older subjects (TILDA, [43]) and in younger adults with vasovagal syncope [44]. However, both studies contained subjects without OH, while we only evaluated symptomatic postural changes in adults with OH. Other studies have shown that patients with orthostatic intolerance had a slower oxygenation recovery rate than healthy adults, and that OH with orthostatic intolerance symptoms presented, on average, with a more pronounced drop in total hemoglobin [45, 46]. As total hemoglobin changes can largely be attributed to changes in oxygenated hemoglobin, this matches our findings.

Daily sit-stand transitions also decreased O_2_Hb and increased HHb in long channels. A sit-stand postural change in accelerometer signals resulted in a recognizable increase and decrease within approximately 2 s. Although most studies use accelerometer data from wrist- (e.g., smartwatches) or body-worn devices, a previous study using accelerometer data from the forehead reported the same pattern [47]. However, individual sit-stand transitions varied much, also within individuals, as these non-standardized movements could be followed or accompanied by walking, squatting, bending, or talking, all affecting oxygenation signals. Therefore, standardized supine-stand tests hold more potential for OH identification. Identifying non-standardized sit-stand transitions in the presence of OH symptoms could provide added value. However, this needs further confirmation as only one participant in our study reported OH symptoms.

### Reliability

Oxygenation parameters showed poor reliability between supine-stand transitions during the laboratory visit and two measurement days at home, with an ICC of maximally 0.40. These ICCs were highest for the maximum drop amplitude, confirming previous literature [20]. Previous research found excellent test–retest reliability (> 0.8) for long-channel oxygenation outcomes (i.e., maximum drop amplitude and late recovery (60–180 s)), except for the late recovery defined as the average of 30–180 s (poor ICC of 0.1) [20, 41]. Inter-observer reliability for long-channel oxygenation outcomes was previously found to be good under standardized laboratory conditions (ICC 0.6) [20]. These oxygenation outcome variables under laboratory conditions indicate that NIRS as a measurement technique is reliable. However, our measurement protocol added sources of variance by measuring in the laboratory and during two distinct days at home and by measuring three times a day a few hours apart. This introduced variation by retesting and reapplying the sensors (sometimes also within a measurement day). Due to the absence of significant differences over measurement settings, low ICCs are likely caused by biological daily signal fluctuations and random variability, such as motion artifacts. Future research, including more repetitions of standardized supine-to-stand transitions over more measurement days, is needed to identify the different sources of variance in play and how to correct for them.

### Clinical implications and recommendations

During the screening laboratory visits, it became apparent that the intention to include distinct groups of participants, i.e., symptomatic with OH and asymptomatic without OH, could not be met. Some participants, previously diagnosed with OH, showed no OH during the screening laboratory visit and were therefore not included in the present study; some of the included participants with OH had periods with symptoms alternating with symptom-free periods, resulting in a low number of symptomatic measurement days in our study. This further emphasizes the heterogeneity of OH and its clinical expression. It also again highlights the shortcomings of using single supine-to-stand BP measurements to identify OH.

Representability of daily life is important for eventual clinical application. During this study, we noticed that participants were hesitant to go outside and were mostly sedentary due to the visibility of the sensor and fear of distorting the measurement. Nevertheless, daily postural changes were still present. Future research including symptomatic OH patients should ensure that (if present) specific activities causing OH-related symptoms are undertaken during the daily NIRS measurement. Moreover, for clinical application, measurements should be as simple as possible, making identification of sit-stand transitions based on accelerometry included in the NIRS sensor alone more desirable than the use of additional devices to detect postural changes (ActivPALS in this study). Future research should therefore investigate the use of sit-stand detection algorithms, for example, based on wavelets, using daily-life IMU data collected on the forehead [47, 48].

### Strengths and limitations

To the best of our knowledge, this is the first study to measure cerebral oxygenation at home during postural changes in participants with OH using NIRS. This study had some limitations. As this was a small-scale study, few participants experienced symptoms during the measurement day, and although the data on this limited number are promising, firm conclusions cannot be drawn regarding validity. Second, we excluded older adults with asymptomatic OH and older adults with impairing orthostatic intolerance symptoms without OH, limiting generalizability. Third, to determine reliability, higher resolution (i.e., more supine-stand repetitions during more measurement days) is necessary.

## Conclusion

This study shows feasibility of NIRS-measured cerebral oxygenation responses to postural changes at home to support the diagnosis and monitoring of OH. Further improvements regarding artifact prevention are desirable however for future individual use. Face validity was shown, as participants with OH had lower oxygenation recovery values after standing than those without OH, whereas symptomatic postural changes showed deeper oxygenation maximum drops after standing than asymptomatic postural changes. These results imply that NIRS holds potential as a monitoring tool for OH at home, which can give insight into the dynamics of OH and response to therapeutic interventions. Further research, including higher-resolution standardized test data, is needed to identify sources of variability, including daily signal fluctuation, and improve reliability of oxygenation measurements at home.

## Supplementary Information

Below is the link to the electronic supplementary material.

Supplementary file1 (DOCX 1795 KB)
