# Supplementary material for: Home-based monitoring of cerebral oxygenation in response to postural changes using near-infrared spectroscopy
Source: GeroScience. 2024 Jun 18;46(6):6331–46. doi: 10.1007/s11357-024-01241-w (PMC11493916; doi:10.1007/s11357-024-01241-w)
Supplement: Supplementary file 1 — Supplementary file1 (DOCX 1795 KB) [file 11357_2024_1241_MOESM1_ESM.docx]

**Supplementary material**

Home-based monitoring of cerebral oxygenation in response to postural changes using near-infrared spectroscopy

**Methods**


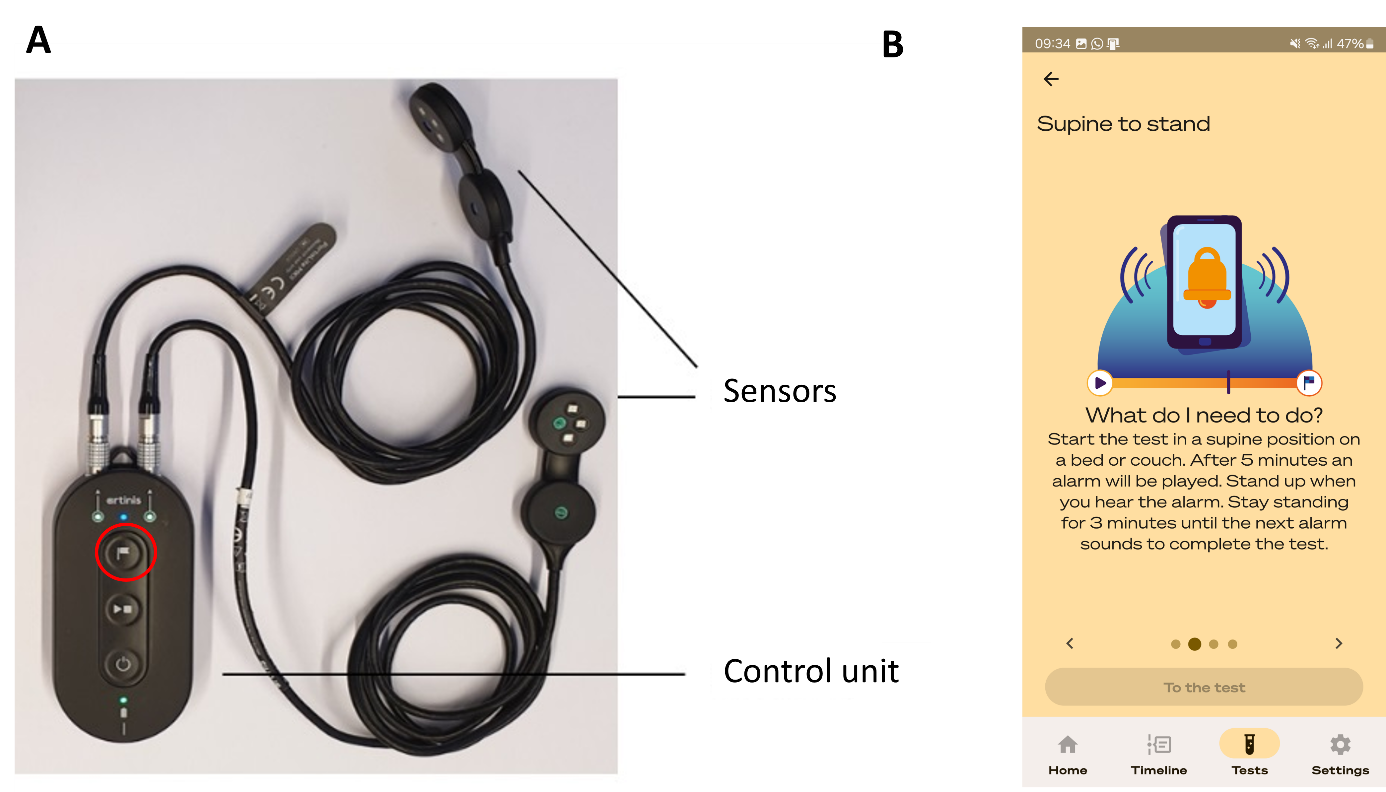


**Fig S.1** Measurement device (PortaLite MkII with sensors and control unit), on which the button to report an event is indicated by a red circle (**A**) and screenshot of smartphone application Krane™ by Orikami, explaining how to perform the supine-stand test (**B**).

**Quality assessment – visualization of SQI**

**Fig. S.2** Visualization of signal quality index (SQI) score per supine-stand repetition for two at-home measurement days. 1 is low quality, and 5 high quality. Short channels are depicted in the lower panels, and long channels in the upper panels. Measurement day 1 is shown on the left, and measurement day 2 on the right.

**OH-related symptoms**

**Fig. S.3** (Cerebral) oxygenation measured with long (**A+C**) and short (**B+D**) channels during standardized supine-stand tests in symptomatic versus asymptomatic postural changes of participants with orthostatic hypotension. Oxygenated hemoglobin is shown in red and deoxygenated in blue. The line represents the mean, and shaded areas the standard deviation.

**Fig. S.4** Three reported 10-minute episodes of OH-related dizziness of one subject, after standing up from sitting during the first measurement day (episode 1 and 2) and after walking during the second measurement day (episode 3). Upper panels show the long-channel oxygenation (blue and red) and lower panels the short-channel oxygenation (blue and red). The green line indicates the accelerometer signal (square root of squared and summed x, y, and z direction, divided by 100 for visualization purposes). The black dashed line indicates the exact moment the event was reported.
